# Health Tract, No. 196

**Published:** 1864-04

**Authors:** 


HEALTH TRACT, No. 196.

### EATING ECONOMICALLY.

One of the good results of the existing civil war will be to inaugurate habits of economy throughout every department of social and domestic life, which will save millions of money every year, so that, in spite of increased taxation, multitudes of careful, thrifty families will be quite as well off as to money matters, as they would have been had there been no war, while, at the same time, they will have acquired a higher moral and social character than they had before, because economy implies carefulness and self-denial, and these are certainly elevating, as we know that waste and self-indulgence degrade, and in the end brutalize as to the appetites and propensities. This is not all. Waste brings want, and want obtunds the moral sense, so that in time it will not only tempt to take mean advantages in business, but next to borrow money, with a consciousness of having no specific means of returning the same; a little later comes deliberate fraud, theft, and robbery outright. To aid the reader in the practice of such high and necessary virtues as carefulness, economy, and a manly self-denial, let a few lessons be taken from an older, more experienced, and wiser nation—the French. The first step for a family to take, especially in New-York, in summer, and in all families where there are no servants, and consequently no need of "keeping up" a kitchen-fire, is to purchase some cooking-lamp for oil or gas, Fish's patent, for example, by which a good meal can be prepared for half a dozen persons for a single cent, this alone will save the price of one or two tons of coal in a year. The older nations do not take any meat for the first meal in the day, we mean the better classes, and those who live mainly in-doors. *Bentley's Miscellany* says of the richer classes of French, that they make an early breakfast of coffee, taking no meat until about noon. They cook no more for one day than lasts that day; and any observant housewife will soon learn how much will be eaten; but if any thing is left over for to-day, less is purchased to-morrow, for waste is not allowed; this saves the wickedness of trying to "eat up" the leavings of the current day. Close observation has shown that, at this time, a French family, in Paris, of three or four persons, with two servants, can live really well, with good management, including ordinary wine, kitchen fuel, and all supplementary expenses for food, for about nine English shillings a day. Outside of Paris, it certainly does not exceed six shillings a day for six persons, or one dollar and a half. This would be a healthier and happier land by far, if parents would make a systematic effort to impress on the minds of their children that waste is an unmitigated wickedness, and that economy is one of the higher virtues, albeit a good many of our children and wives consider it "mean," and are absolutely ashamed that their unprincipled and cribbing servants should think they were trying to economize. Millions of money could be saved every year, if the larger cities of this country could adopt the plan of many Europeans, have no cooking done in the house, except for making a cup of tea or coffee, toasting bread, and boiling a potato, all of which a lamp can do, having other things prepared at the public cookeries. In other words, have dinner prepared outside, to be kept on the table "smoking hot," if desired, by means of little lamps. This plan works well abroad, could be made to work acceptably here and would save a large per centage of the cost of housekeeping.

